# Inflammation, infection, and immune dysregulation in chronic kidney disease: translational and epidemiological perspectives

**DOI:** 10.3389/fneph.2026.1919658

**Published:** 2026-07-22

**Authors:** Aminu Shittu

**Affiliations:** Department of Veterinary Public Health and Preventive Medicine, Faculty of Veterinary Medicine, Usmanu Danfodiyo University, Sokoto, Nigeria

**Keywords:** biomarkers, chronic kidney disease, immune dysregulation, infection, inflammation, precision medicine, renal immune microenvironment, translational nephrology

## Abstract

Chronic kidney disease (CKD) represents a growing global health challenge associated with substantial morbidity, mortality, and healthcare burden. Although metabolic and haemodynamic factors, particularly diabetes mellitus and hypertension, remain major contributors, increasing evidence demonstrates that persistent inflammation and immune dysregulation are central mechanisms influencing CKD initiation, progression, and complications. The renal immune microenvironment consists of complex interactions among resident kidney cells, infiltrating immune cells, inflammatory mediators, and molecular signalling networks that regulate tissue repair, fibrosis, and disease outcomes. Persistent activation of innate and adaptive immune responses promotes cytokine release, oxidative stress, endothelial dysfunction, and maladaptive tissue remodelling, contributing to progressive loss of kidney function. Infectious diseases and altered host–microbiome interactions may further amplify systemic inflammation and immune imbalance, particularly in vulnerable populations. Advances in immunology and molecular medicine have identified inflammatory biomarkers and immune-related pathways with potential applications in early detection, risk stratification, and targeted interventions. This Mini Review synthesizes current evidence linking inflammation, infection, and immune dysregulation with CKD progression, highlighting translational opportunities and epidemiological perspectives. Integrating mechanistic insights with population-level evidence may accelerate precision approaches for improving CKD prevention, monitoring, and therapeutic outcomes.

## 1 Introduction

Chronic kidney disease (CKD) represents a major global health challenge, affecting more than 850 million people worldwide and contributing substantially to morbidity, mortality, reduced quality of life, and healthcare expenditure (1, 2). Although CKD progression has traditionally been attributed to metabolic and haemodynamic disturbances, particularly diabetes mellitus and hypertension, growing evidence indicates that persistent inflammation and immune dysregulation are fundamental contributors to kidney injury and progressive functional decline (3, 4).

The kidney is increasingly recognized as an immunologically active organ where interactions among resident renal cells, infiltrating immune populations, inflammatory mediators, and molecular signalling pathways determine the balance between tissue repair and chronic injury (5). Persistent immune activation following metabolic stress, infection, cellular injury, or environmental exposures can promote oxidative stress, cytokine release, endothelial dysfunction, and progressive fibrosis (6, 7). This evolving understanding has shifted perspectives of CKD from a primarily structural disorder towards a complex immune-inflammatory condition with important implications for biomarker discovery and targeted interventions.

Infectious diseases and chronic inflammatory states may further influence immune responses, accelerate renal injury, and contribute to disparities in CKD outcomes, particularly in populations with high infectious disease burdens and limited healthcare resources (8, 9). This Mini Review synthesizes current evidence linking inflammation, infection, and immune dysregulation with CKD progression, highlighting emerging biomarkers, therapeutic opportunities, and epidemiological perspectives relevant to translational nephrology.

## 2 Renal immune microenvironment overview

The renal immune microenvironment represents a dynamic network of resident kidney cells, innate and adaptive immune populations, soluble mediators, and extracellular matrix components that collectively regulate tissue homeostasis and responses to injury (10, 11). Once considered mainly a passive target of immune-mediated damage, the kidney is now recognized as an active immunological organ where immune and non-immune cellular interactions influence the transition from adaptive repair to progressive disease (12, 13).

Innate immune cells, including macrophages, dendritic cells, neutrophils, and natural killer cells, represent early responders to tissue stress and injury signals. Activation of pattern-recognition receptors, such as Toll-like receptors and inflammasome pathways, promotes cytokine and chemokine production that coordinates local immune responses (14, 15). Although these mechanisms are essential for host defence and tissue repair, persistent activation contributes to chronic inflammation, tubular damage, and fibrotic remodelling (6, 16).

Adaptive immunity also influences CKD pathogenesis through interactions involving T lymphocyte subsets, B cells, and regulatory immune pathways. Imbalance between pro-inflammatory and regulatory responses may sustain immune activation and accelerate progressive nephron loss (12, 17). Therefore, understanding the renal immune microenvironment provides a framework for identifying mechanisms driving CKD progression and developing novel diagnostic and therapeutic strategies.

## 3 Chronic inflammation and CKD progression

Persistent low-grade inflammation is increasingly recognized as a key mechanism linking renal injury with progressive kidney dysfunction. Unlike acute inflammatory responses that facilitate repair, unresolved inflammation promotes sustained immune activation, excessive cytokine production, oxidative stress, and extracellular matrix accumulation, ultimately contributing to glomerular dysfunction, tubular injury, and renal fibrosis (18, 19).

Several interconnected molecular pathways regulate inflammation-driven CKD progression. Activation of nuclear factor-kappa B (NF-κB) signalling promotes expression of inflammatory mediators, including interleukin-6 (IL-6), tumour necrosis factor-alpha (TNF-α), and chemokines involved in immune-cell recruitment and tissue injury (18, 20). Similarly, transforming growth factor-beta (TGF-β) contributes to fibrogenesis through fibroblast activation and extracellular matrix deposition, leading to structural remodelling of renal tissues (21).

Oxidative stress further amplifies renal injury by disrupting cellular function, enhancing inflammatory signalling, and promoting endothelial and mitochondrial dysfunction (22). Declining kidney function may also increase accumulation of uraemic toxins, creating a self-perpetuating cycle of immune activation and progressive damage (23).

In addition to innate inflammatory signalling, adaptive immune responses also contribute substantially to CKD progression. T-helper (Th) cell subsets, particularly Th1 and Th17 cells, amplify renal inflammation through the production of pro-inflammatory cytokines, including interferon-γ (IFN-γ) and interleukin-17 (IL-17), thereby promoting leukocyte recruitment, tubular injury, and progressive fibrosis. In contrast, regulatory T cells (Tregs) help preserve immune homeostasis by suppressing excessive inflammatory responses through anti-inflammatory mediators such as interleukin-10 (IL-10) and transforming growth factor-β (TGF-β). An imbalance favouring Th1/Th17 responses over Treg activity has been implicated in sustained immune activation, progressive nephron loss, and worsening renal function, underscoring the important contribution of adaptive immunity to the renal immune microenvironment and its potential as a target for future immunomodulatory therapies (12, 13, 17, 18).

**Figure 1:**
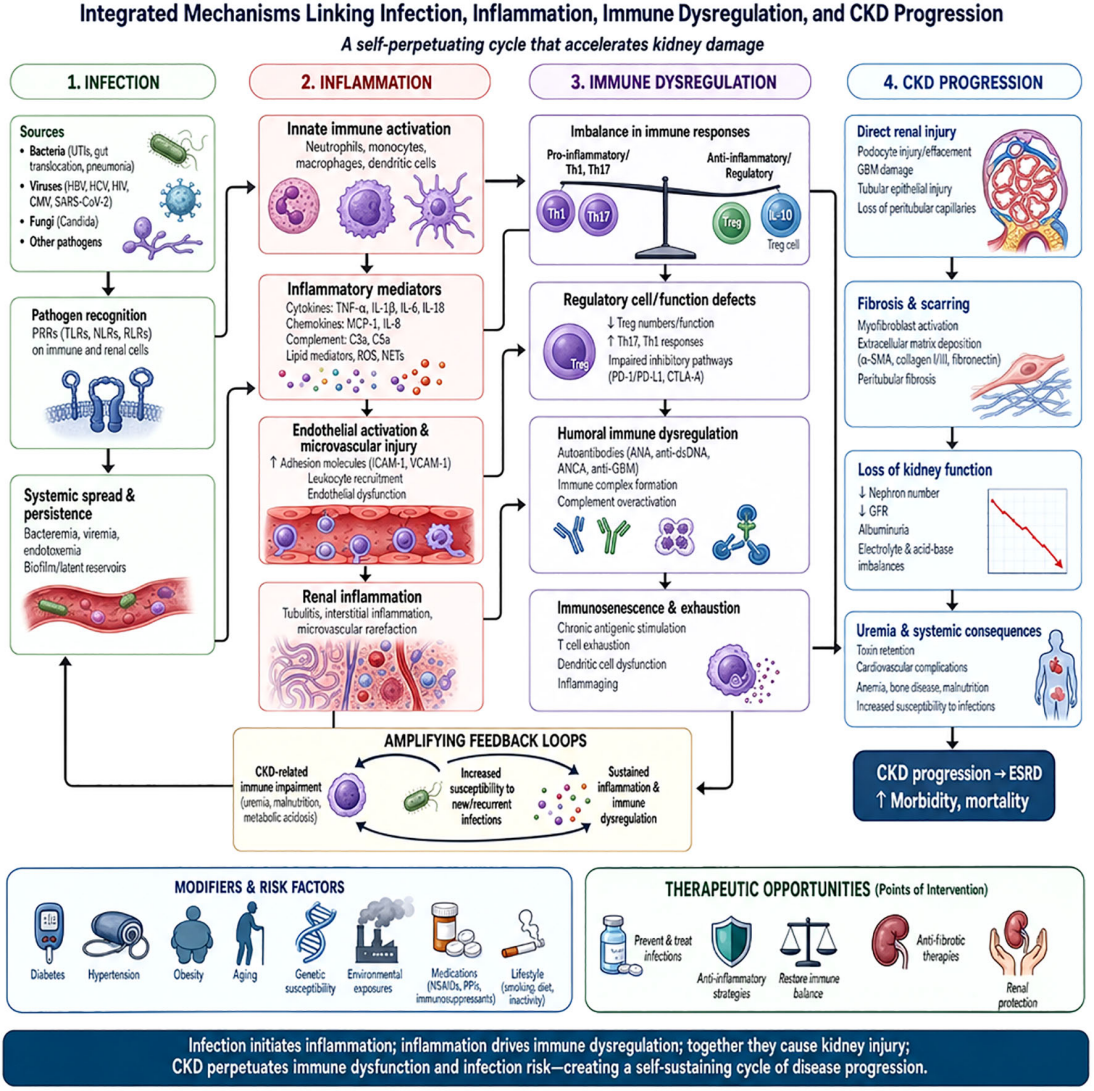
Integrated mechanisms linking infection, inflammation, immune dysregulation, and chronic kidney disease progression.

The interactions among inflammatory triggers, immune pathways, and CKD progression are summarized in **Figure 1**.

## 4 Infectious diseases and renal immune dysregulation

Infectious diseases represent important external drivers of immune activation and may contribute to kidney injury through direct pathogen-related effects, persistent inflammation, and dysregulated host responses. Recognition of microbial pathogen-associated molecular patterns (PAMPs) by pattern-recognition receptors, particularly Toll-like receptors (TLRs), activates macrophages and dendritic cells, leading to enhanced production of pro-inflammatory cytokines, including interleukin-1β (IL-1β), interleukin-6 (IL-6), and tumour necrosis factor-α (TNF-α). This inflammatory cascade further promotes Th1 and Th17 immune responses, amplifies oxidative stress, endothelial dysfunction, and fibroblast activation, and ultimately accelerates renal fibrosis and progressive nephron loss, thereby providing a mechanistic link between infection and the broader immune pathways underlying CKD progression, as illustrated in **Figure 1** (12–14, 18, 20). During infection, activation of innate immune pathways promotes inflammatory signalling required for pathogen clearance; however, excessive or prolonged activation may disrupt renal homeostasis and contribute to acute and chronic kidney injury (18, 24).

Several infections have been associated with renal complications through immune-mediated mechanisms. Viral infections, including human immunodeficiency virus (HIV) and hepatitis viruses, may promote glomerular injury, immune-complex deposition, and progressive renal dysfunction (25, 26). Similarly, bacterial infections and sepsis can induce systemic inflammatory responses involving endothelial dysfunction, complement activation, and immune-cell infiltration, potentially contributing to the transition from acute kidney injury to CKD (27, 28).

Beyond individual pathogens, emerging evidence suggests that alterations in host–microbiome interactions may influence renal immune regulation. Gut dysbiosis in CKD has been associated with accumulation of uraemic toxins, systemic inflammation, and disruption of immune homeostasis (29, 30). Understanding these infection–immunity–kidney interactions is particularly relevant in populations experiencing high infectious disease burdens and limited access to early kidney care.

## 5 Antimicrobial resistance and kidney complications

Antimicrobial resistance (AMR) represents an emerging challenge connecting infectious diseases, therapeutic limitations, and kidney health. Individuals with CKD are at increased risk of infections due to impaired immune responses, frequent healthcare exposure, and underlying comorbidities, often requiring repeated antimicrobial interventions that may increase selection pressure for resistant pathogens (31, 32).

The relationship between antimicrobial therapy and kidney injury remains clinically important. Certain antimicrobial agents, including aminoglycosides and glycopeptides, may contribute to nephrotoxicity through tubular injury, oxidative stress, and inflammatory mechanisms, creating challenges when treating infections in patients with reduced renal function (33, 34).

Integrating antimicrobial stewardship, infection prevention, and renal-protective strategies through a One Health approach may therefore help reduce infection-related kidney complications and improve outcomes among vulnerable populations.

## 6 Biomarkers and immune pathways

Advances in understanding immune-inflammatory mechanisms in CKD have increased interest in identifying biomarkers that reflect disease activity, predict progression, and guide therapeutic strategies. Although traditional measures such as serum creatinine and estimated glomerular filtration rate (eGFR) remain central to clinical evaluation, they provide limited information on early molecular alterations preceding irreversible kidney damage (35–38). Therefore, biomarkers reflecting inflammation, tubular injury, and immune activation may improve early detection and individualized risk assessment.

Systemic inflammatory mediators, including C-reactive protein (CRP), interleukin-6 (IL-6), and tumour necrosis factor-alpha (TNF-α), have been associated with inflammatory burden, cardiovascular complications, and adverse outcomes in CKD (39, 40). Similarly, kidney-specific biomarkers such as neutrophil gelatinase-associated lipocalin (NGAL) and kidney injury molecule-1 (KIM-1) provide insights into tubular injury and cellular stress responses that may occur before advanced functional decline (41, 42).

Beyond individual biomarkers, immune pathways involving inflammasome activation, complement signalling, and fibrotic mediators are increasingly recognized as potential therapeutic targets (43). Integration of biomarker profiling with molecular and clinical data may advance precision nephrology by enabling improved patient stratification and targeted interventions.

Key immune-inflammatory biomarkers and emerging therapeutic targets relevant to CKD are summarized in **Table 1**.

**Table 1:** Emerging immune-inflammatory biomarkers and therapeutic targets in chronic kidney disease: biological functions, clinical implementation, and translational relevance.

Biomarker/pathway	Biological function	Role in CKD progression	Clinical implementation and translational relevance	Representative references
C-reactive protein (CRP)	Acute-phase inflammatory protein mainly induced by IL-6 signalling during systemic inflammation	Reflects persistent low-grade inflammation associated with endothelial dysfunction, cardiovascular complications, accelerated CKD progression, and increased mortality risk	Routine clinical biomarker for inflammatory burden, risk stratification, and monitoring systemic inflammatory status in CKD patients	(18, 37, 38, 40)
Interleukin-6 (IL-6)	Pro-inflammatory cytokine involved in immune-cell activation, acute-phase responses, and regulation of inflammatory signalling	Promotes chronic inflammation, endothelial dysfunction, oxidative stress, vascular injury, and cardiovascular complications associated with CKD	Primarily a research biomarker, with potential utility for disease severity assessment and cytokine-targeted therapies	(18, 37, 39, 40)
Tumour necrosis factor-alpha (TNF-α)	Major inflammatory cytokine regulating immune activation, apoptosis, and inflammatory cell recruitment	Enhances oxidative stress, tubular injury, endothelial activation, and amplification of inflammatory responses within renal tissues	Experimental therapeutic target, with potential application in limiting immune-mediated renal injury	(18, 38–40)
Interleukin-1β (IL-1β)	Inflammasome-dependent cytokine involved in innate immune activation and inflammatory amplification	Promotes inflammatory signalling, immune-cell recruitment, and tissue injury during persistent renal inflammation	Investigational therapeutic target for inflammasome- directed immunomodulation	(14, 15, 24, 43)
Transforming growth factor-beta (TGF-β)	Regulates tissue repair, extracellular matrix production, and fibroblast activation	Major driver of renal fibrosis through myofibroblast activation, extracellular matrix accumulation, and structural remodelling	Established experimental therapeutic target for anti-fibrotic interventions aimed at slowing CKD progression	(6, 16, 21)
Nuclear factor-kappa B (NF-κB)	Transcription factor regulating expression of inflammatory genes and immune mediators	Sustained activation increases cytokine production, immune-cell infiltration, and chronic renal inflammation	Experimental molecular target for controlling maladaptive inflammatory responses	(6, 18, 20)
NLRP3 inflammasome	Innate immune sensor complex regulating inflammatory cytokine maturation and immune activation	Activation promotes IL-1β release, tubular injury, oxidative stress, and renal fibrosis	Emerging therapeutic target, with several immunomodulatory strategies currently under investigation	(14, 19, 24, 43)
Complement system (C3/C5 pathways)	Innate immune defence mechanism involved in pathogen recognition and immune regulation	Excessive activation contributes to glomerular inflammation, immune-mediated injury, and progressive kidney damage	Clinically actionable pathway, with complement inhibitors already available for selected immune-mediated kidney diseases	(5, 13, 43, 57)
Monocyte chemoattractant protein-1 (MCP-1/CCL2)	Chemokine regulating recruitment of monocytes/macrophages to sites of renal injury	Promotes macrophage infiltration, persistent inflammation, and fibrotic responses in kidney tissues	Emerging biomarker and therapeutic target for inflammatory kidney diseases	(6, 7, 18)
Reactive oxygen species (ROS)/oxidative stress pathways	Regulate cellular signalling but cause cellular injury when excessively produced	Induces mitochondrial dysfunction, endothelial injury, inflammation, and progressive nephron damage	Experimental therapeutic pathway supporting antioxidant and mitochondrial-targeted interventions.	(4, 6, 22)
Neutrophil gelatinase-associated lipocalin (NGAL)	Protein released by injured tubular epithelial cells during cellular stress	Indicates tubular injury, inflammation, and early kidney damage before advanced functional decline	Emerging clinical biomarker for early detection of kidney injury and prediction of renal outcomes	(35, 36, 41, 42)
Kidney injury molecule-1 (KIM-1)	Transmembrane protein expressed by damaged proximal tubular epithelial cells	Reflects tubular epithelial injury, repair responses, and ongoing kidney damage	Emerging clinical biomarker for detecting tubular injury and monitoring disease activity	(35, 36, 41, 42)
Soluble urokinase plasminogen activator receptor (suPAR)	Circulating immune-derived protein reflecting immune activation and inflammation	Associated with immune dysfunction, podocyte injury, and risk of kidney function decline	Investigational biomarker for CKD prediction and risk stratification that has not yet entered routine clinical practice	(35–37)
Fibroblast growth factor-23 (FGF-23)	Hormone involved in phosphate regulation and mineral metabolism	Elevated levels contribute to CKD-mineral bone disorder, inflammation, and cardiovascular complications	Clinically useful risk marker for CKD-mineral bone disorder and cardiovascular complications	(35, 36, 56)
Uraemic toxins (e.g., indoxyl sulfate, p-cresyl sulfate)	Metabolic products accumulating due to reduced renal clearance	Promote oxidative stress, inflammation, immune dysfunction, and cardiovascular injury	Emerging therapeutic targets through toxin reduction, microbiome modulation, and metabolic interventions	(23, 29, 30)
Gut microbiome-related pathways	Regulate host metabolism, immune homeostasis, and host–microbial interactions	Dysbiosis contributes to uraemic toxin generation, systemic inflammation, and immune dysregulation	Experimental therapeutic target, with dietary and microbiome-based interventions under active investigation	(23, 29, 30)
Immune-cell profiling (macrophage/T-cell phenotypes)	Reflects balance between protective and pathogenic immune responses	Altered macrophage and lymphocyte responses contribute to inflammation, fibrosis, and impaired repair	Future precision nephrology approach supporting individualized immune-based therapies and patient stratification	(7, 11, 13, 17, 58, 59)

CKD, chronic kidney disease; CRP, C-reactive protein; FGF-23, fibroblast growth factor-23; IL, interleukin; KIM-1, kidney injury molecule-1; MCP-1, monocyte chemoattractant protein-1; NF-κB, nuclear factor-kappa B; NGAL, neutrophil gelatinase-associated lipocalin; NLRP3, nucleotide-binding oligomerization domain-like receptor family pyrin domain-containing 3; ROS, reactive oxygen species; suPAR, soluble urokinase plasminogen activator receptor; TGF-β, transforming growth factor-beta; TNF-α, tumour necrosis factor-alpha.

## 7 Epidemiological and global burden evidence

The increasing global burden of CKD emphasizes the importance of integrating mechanistic discoveries with population-level evidence to improve prevention and clinical outcomes. CKD prevalence, risk factors, and access to diagnosis and treatment vary substantially across geographical regions and socioeconomic settings (44, 45). Although diabetes mellitus and hypertension remain dominant contributors, non-traditional factors, including chronic infections, environmental exposures, and inflammatory conditions, may also influence kidney disease patterns, particularly in low- and middle-income countries (46, 47).

Epidemiological approaches provide opportunities to understand how immune-inflammatory mechanisms interact with broader determinants of kidney health. Integrating population-based studies with immunological profiling and biomarker discovery could improve identification of vulnerable populations, enhance risk prediction, and support targeted prevention strategies (48). Such approaches may help translate advances in renal immune microenvironment research into equitable improvements in global kidney health.

## 8 Translational and therapeutic implications

Improved understanding of the renal immune microenvironment has created opportunities for therapeutic strategies that extend beyond conventional management of established CKD risk factors. Although interventions targeting blood pressure control, glycaemic regulation, and renin–angiotensin–aldosterone system modulation remain fundamental, many patients continue to experience progressive kidney dysfunction, emphasizing the need for approaches targeting underlying inflammatory and immune mechanisms (49, 50).

Emerging strategies increasingly focus on modulation of immune signalling pathways involved in renal inflammation, oxidative injury, and fibrosis. Targeting cytokine networks, complement activation, inflammasome signalling, and profibrotic pathways may provide opportunities to limit maladaptive immune responses and preserve kidney function (51, 52). In addition, recent advances with sodium–glucose cotransporter-2 (SGLT2) inhibitors demonstrate that therapies originally developed for metabolic regulation may exert broader kidney-protective effects partly through modulation of inflammation, oxidative stress, and cellular injury pathways (53–55).

Future translational nephrology will increasingly rely on integrating immune biomarkers, molecular profiling, and clinical information to support precision approaches. Combining mechanistic discoveries with epidemiological insights may enable earlier disease detection, improved patient stratification, and development of targeted interventions to reduce CKD progression.

## 9 Research gaps and future directions

Despite growing recognition of immune-inflammatory mechanisms in CKD, important knowledge gaps remain. Many discoveries regarding renal immune pathways originate from experimental models or selected patient populations, emphasizing the need for large-scale longitudinal studies integrating molecular, clinical, and epidemiological data across diverse populations (56, 57).

Future research should prioritize validation of immune-based biomarkers, identification of patient-specific inflammatory signatures, and evaluation of targeted interventions that modify disease progression. Integration of multi-omics technologies, artificial intelligence, and precision medicine frameworks may improve understanding of CKD heterogeneity and support individualized approaches to prevention and treatment (36, 58, 59). Greater inclusion of populations from low-resource settings will also be essential to understand how infections, environmental exposures, and healthcare inequalities influence immune-mediated kidney disease.

## 10 Conclusion

CKD is increasingly recognized as a complex immune-inflammatory disorder influenced by interactions among metabolic disturbances, infections, environmental factors, and the renal immune microenvironment. Persistent immune activation contributes to progressive kidney injury, while advances in biomarker discovery and targeted therapies offer new opportunities for improved management. Bridging mechanistic understanding with epidemiological evidence will be essential for translating discoveries into effective clinical interventions. Multidisciplinary approaches integrating nephrology, immunology, infectious disease research, and public health may support more precise and equitable strategies for reducing the global burden of CKD. Future integration of immune profiling, population-level evidence, and targeted therapeutic strategies may accelerate the transition towards precision nephrology.
